# ART adherence and viral suppression are high among most non‐pregnant individuals with early‐stage, asymptomatic HIV infection: an observational study from Uganda and South Africa

**DOI:** 10.1002/jia2.25232

**Published:** 2019-02-12

**Authors:** Jessica E Haberer, Bosco M Bwana, Catherine Orrell, Stephen Asiimwe, Gideon Amanyire, Nicholas Musinguzi, Mark J Siedner, Lynn T Matthews, Alexander C Tsai, Ingrid T Katz, Kathleen Bell, Annet Kembabazi, Stephen Mugisha, Victoria Kibirige, Anna Cross, Nicola Kelly, Bethany Hedt‐Gauthier, David R Bangsberg

**Affiliations:** ^1^ Massachusetts General Hospital Center for Global Health Boston MA USA; ^2^ Harvard Medical School Boston MA USA; ^3^ Mbarara University of Science and Technology Mbarara Uganda; ^4^ Global Health Collaborative Mbarara Uganda; ^5^ Desmond Tutu HIV Foundation Cape Town South Africa; ^6^ University of Cape Town Cape Town South Africa; ^7^ Kabwohe Clinical Research Centre Kabwohe Uganda; ^8^ Makerere University Joint AIDS Program Kampala Uganda; ^9^ Africa Health Research Institute Durban South Africa; ^10^ Brigham and Women's Hospital Boston MA USA; ^11^ Oregon Health & Science University‐Portland State University School of Public Health Portland OR USA

**Keywords:** HIV, antiretroviral therapy, adherence, stage of disease, sub‐Saharan Africa

## Abstract

**Introduction:**

The success of universal antiretroviral therapy (ART) access and aspirations for an AIDS‐free generation depend on high adherence in individuals initiating ART during early‐stage HIV infection; however, adherence may be difficult in the absence of illness and associated support.

**Methods:**

From March 2015 to October 2017, we prospectively observed three groups initiating ART in routine care in Uganda and South Africa: men and non‐pregnant women with early‐stage HIV infection (CD4 > 350 cells/μL), pregnant women with early‐stage HIV infection and men and non‐pregnant women with late‐stage HIV infection (CD4 < 200 cells/μL). Socio‐behavioural questionnaires were administered and viral loads were performed at 0, 6 and 12 months. Adherence was monitored electronically.

**Results:**

Adherence data were available for 869 participants: 322 (37%) early/non‐pregnant, 199 (23%) early/pregnant and 348 (40%) late/non‐pregnant participants. In Uganda, median adherence was 89% (interquartile range 74 to 96) and viral suppression was 90% at 12 months; neither differed among groups (*p* > 0.72). In South Africa, median adherence was higher in early/non‐pregnant versus early/pregnant or late/non‐pregnant participants (76%, 37%, 52%; *p* < 0.001), with similar trends in viral suppression (86%, 51%, 79%; *p* < 0.001). Among early/non‐pregnant individuals in Uganda, adherence was higher with increasing age and lower with structural barriers; whereas in South Africa, adherence was higher with regular income, higher perceived stigma and use of other medications, but lower with maladaptive coping and cigarette smoking.

**Discussion:**

ART adherence among non‐pregnant individuals with early‐stage infection is as high or higher than with late‐stage initiation, supporting universal access to ART. Challenges remain for some pregnant women and individuals with late‐stage infection in South Africa and highlight the need for differentiated care delivery.

## 1 Introduction

Global aspirations for an AIDS‐free generation are inspired by data showing that antiretroviral therapy (ART)‐mediated HIV viral suppression reduces HIV transmission risk by 96% 1, 2, and recent studies provide strong evidence that undetectable = untransmissible (U = U) 3, 4. Moreover, two randomized controlled trials demonstrated wide‐ranging health benefits of immediate versus delayed ART, even at relatively high CD4 counts 5, 6. These findings have led to World Health Organization (WHO) treatment guidelines to initiate ART for all people living with HIV (PLWH) regardless of CD4 count 7. Notably, these guidelines assume that adherence does not vary by HIV disease stage at ART initiation. If patients with early‐stage disease have low adherence, ART expansion could be accompanied by high levels of viraemia, poor health, drug resistance, and/or increased secondary HIV transmission 8 ‐ all of which would mitigate the clinical and preventive benefits of early ART.

ART adherence during late‐stage HIV disease has typically been high in sub‐Saharan Africa among those engaged in care and has been thought to be driven largely by social network activation to overcome adherence barriers 9. Social support helps PLWH overcome structural and economic barriers to adherence (e.g. living in geographically remote areas and needing to pay high transportation costs to pick up ART) 10, 11. Access to social support, however, requires HIV status disclosure, which may be less common in early‐stage disease due to HIV stigma 12, 13, 14, 15. These issues may be particularly relevant during pregnancy and post‐partum when numerous social and biological stressors may converge 16. Moreover, illness is often a strong catalyst for encouraging HIV disclosure, which enables PLWH to access the support needed to achieve high ART adherence. Yet, symptomatic illness is less prominent for individuals with early‐stage HIV infection 16, 17.

Data are sparse on adherence and treatment outcomes among people presenting early to care in programmatic settings, partially because of the persistence of late presentation to care and treatment initiation and particularly in sub‐Saharan Africa 18, 19, 20. A recently published systematic review and meta‐analysis found that patients with higher (vs. lower) CD4 counts were less likely to achieve excellent adherence, although many studies found no difference between these groups 21. Importantly, adherence monitoring was limited to self‐report and/or pharmacy data in all studies and none of the six prospective studies involved patient follow up after 2010, when the recommended CD4 count threshold for initiating treatment increased to 350 cells/μL. The meta‐analysis authors therefore called for additional high‐quality studies, particularly among adults initiating ART at higher CD4 cell counts.

In this study, we prospectively observed three groups of individuals initiating ART in routine care in southwestern Uganda and Cape Town, South Africa over 12 months, using electronic adherence monitors to provide a detailed, objective assessment of adherence behaviour. The groups included men and non‐pregnant women with early‐stage HIV infection (CD4 > 350 cells/μL); pregnant women with early‐stage HIV infection; and men and non‐pregnant women with late‐stage HIV infection (CD4 < 200 cells/μL). Here, we present the levels of adherence and viral suppression among these groups with a goal of testing our hypothesis that ART adherence may be lower in individuals with early‐stage HIV infection at ART initiation (with and without pregnancy) compared to those with late‐stage infection. Among those with early‐stage HIV infection, we also explore which socio‐behavioural factors may influence their adherence.

## 2 Methods

### 2.1 Study settings

In Uganda, participants were recruited from the Mbarara Regional Referral Hospital, Mbarara Municipal Council Health Centre IV, Kabwohe Clinical Research Centre, Kabwohe Health Centre IV and Health Center III facilities in Mushanga, Kakoba and Nyamitanga. All are located approximately 275 km from Kampala in a largely rural area, near a peri‐urban centre in Mbarara Town; HIV prevalence is approximately 8%. In South Africa, participants were recruited from the Hannan Crusaid Treatment Centre and Vuyani, NY1 and Nyanga Clinics. All are located in Gugulethu ‐ a township near Cape Town, South Africa with high levels of poverty; HIV prevalence is approximately 17%. An estimated 78% of all individuals in care in both Uganda and South Africa are virally suppressed 22.

### 2.2 Participants

Individuals were recruited between March 2015 and September 2016 into one the following three groups using convenience sampling as they presented for HIV care:

1. “Early/non‐pregnant”: men and non‐pregnant women living with HIV and initiating ART with asymptomatic early‐stage HIV infection (CD4 > 350 cells/μL; WHO stage I)
2. “Early/pregnant”: pregnant women living with HIV and initiating ART with asymptomatic early‐stage HIV infection (CD4 > 350 cell/μL; WHO stage I)
3. “Late/non‐pregnant”: men and non‐pregnant women living with HIV and initiating ART with late‐stage HIV infection (CD4 < 200 cells/μL).

Inclusion criteria were being ART‐naïve and initiating ART within 28 days of enrolment, >18 years old, living within 60 km of the clinic, and intending to stay in the area for one year. We did not enrol individuals with CD4 counts between 200 and 350 cells/μL because immunological status in this range has less clear associations with health compared to lower and higher CD4 counts. A “late/pregnant” group was not enrolled due to limited resources. Pregnant participants had to be <34 weeks in pregnancy per best available estimates at enrolment. The only exclusion criteria were cognitive impairment (e.g. intoxication or psychosis) such that informed consent could not be obtained or the inability to communicate in English or the primary local languages (i.e. Runyankole in Uganda and Xhosa in South Africa).

### 2.3 Study procedures

Participants received routine care at their local clinics. Standard first‐line ART (once daily tenofovir/emtricitabine/efavirenz in a single pill) was provided by the study to avoid variations in adherence due to regimen stock outs; changes in ART were made per routine clinical guidance. Participants were seen for study visits at baseline, 6 and 12 months. Contact between study staff and participants in between visits was infrequent, by design, to minimize influence of the study on participant behaviour and only occurred to troubleshoot technical problems with the adherence monitors. At study visits, participants provided blood for viral load (determined by Cobas Taqman Test in Uganda and Roche CAP/CTM HIV‐1 v2 assay in South Africa) and research assistants administered surveys on handheld tablets. All materials were presented in the participant's preferred language (i.e. English, Runyankole, or Xhosa), and accuracy of translations were confirmed with back translations. Socio‐behavioural factors potentially relevant for adherence were chosen based on the Behavioral Model for Vulnerable Populations 23 and included socio‐demographics, structural barriers to care (scale of 0 to 52, higher score indicating more barriers 24), severe food insecurity (Household Food Insecurity Access Scale 25), HIV stigma (scale 1 to 4, higher score indicating more stigma, adapted Berger HIV Stigma Scale 26), HIV disclosure, coping (scale of 1 to 4, higher score indicating maladaptive coping 27), medical mistrust/conspiracy (scale 1 to 4, higher score indicating more mistrust/conspiracy 28), necessity and concerns about taking ART 29, clinic satisfaction (scale 1 to 4, higher score indicating more satisfaction; adapted from 30), mental and physical health (Medical Outcomes Study‐HIV, normalized with 50 indicating average health in a U.S. population 31), depression symptom severity (Hopkins Symptom Checklist, probable depression indicated by an average score of >1.75 32) and alcohol use (AUDIT‐C 33). The degree of emotional or instrumental support received from social network ties was assessed by using name generator questionnaires to elicit up to 20 ties, each of whom were rated for the degree of support provided to the participant 34. The social support score reflects the proportion of each participant's supporters at each study visit who were categorized as completely supportive. Adherence was monitored with a real‐time electronic adherence pill container. Each time the monitor was opened for presumed medication ingestion, a date‐and‐time stamp was recorded and transmitted over the cellular network; the monitor additionally sent a daily “heartbeat” to indicate device functionality (Wisepill Technologies, South Africa). Research assistants explained the purpose and function of the device to participants at enrolment, asking them to store only their ART in the device and remove one dose at a time. If participants were hesitant to use the monitor (e.g. due to travel), they were advised to use an alternate pill container during those periods.

### 2.4 Statistical analysis

Participant characteristics were summarized descriptively. Comparisons between sites were made with Wilcoxson rank sum and Pearson's chi‐square tests for continuous and categorical variables respectively. Adherence was calculated as the number of adherence monitor opening events received divided by the number of monitor opening events expected during follow up. Adherence was capped at 100% per day and censored at death. Adherence for participants lost to follow up (defined as no contact despite multiple attempts through 13 months after enrolment) was calculated until the device stopped transmitting data or until the last scheduled study visit ‐ whichever came first. Monitor opening events were removed from the data set for staff openings and during periods of known device malfunction. A secondary data set was created in which device non‐use as reported by participants was also removed. Adherence interruptions of 7+ days were assessed, as this duration of non‐adherence has been associated with >5% risk for viraemia 35. Participants were considered viraemic if they died or were lost to follow up, or missed visits and had <80% adherence in the month before the visit. If a visit was missed in South Africa, available viral load data within one month of the visit were obtained through the National Health Laboratory Service, a previously validated approach 36.

Adherence distributions between groups were compared with the Kruskal Wallis test. Viral suppression was defined as <400 copies/mL and compared among groups by Pearson's chi‐square test. Changes over time were assessed by conditional linear regression. A pre‐planned interim analysis was conducted once half of participants reached the 6‐month time point to determine the need for early termination of the study; no comparisons met the pre‐specified criteria. To test our hypothesis that adherence may differ by HIV stage at ART initiation, we ran linear generalized estimating equation (GEE) regression models; cluster‐correlated robust estimates of variance were used to account for within‐participant dependence of observations. Confounders were identified by a significance level of *p* < 0.10 in univariable models and retained in multivariable models. Uganda and South Africa sites were analysed separately because of numerous demographic and socio‐economic differences between them. Because of collinearity, emotional social support was removed and instrumental social support was retained. We ran similar linear GEE models to assess for adherence predictors among non‐pregnant individuals with early‐stage HIV infection. All analyses were conducted in Stata 13 (StataCorp., College Station, TX, USA).

### 2.5 Ethics

This study was approved by all relevant institutional review boards: Partners Healthcare/Massachusetts General Hospital, Mbarara University of Science and Technology, Uganda National Council for Science and Technology, University of Cape Town and Western Cape province in South Africa. All participants provided written informed consent.

## 3 Results

### 3.1 Participant characteristics

**Figure 1:**
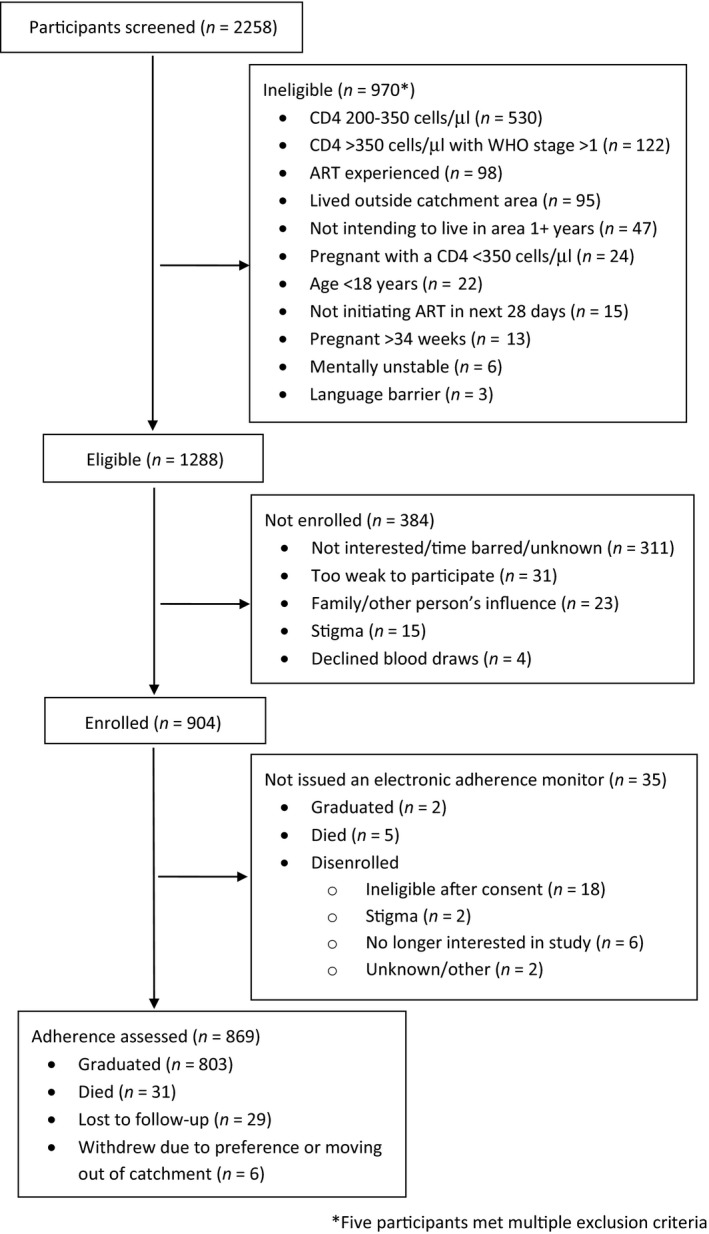
Flow diagram of participant screening, enrolment and follow‐up

We screened 2258 participants and enrolled 904 between March 2015 and September 2016, including 483 in Uganda and 421 in South Africa (Figure 1). The most common reason for exclusion was a CD4 count between 200 and 350  cells/μL (n = 530, 55%). Seventeen percent of screened participants (n = 384) were eligible, but did not enrol; no differences in age, gender or study group were seen in those who did not versus did enrol in South Africa. However, in Uganda, those who did not enrol were more likely to be male (42% vs. 31%, *p* = 0.002). The distribution across some study groups also differed, comparing those who did not versus did enrol: early/non‐pregnant group (39% vs. 37%; *p* = 0.62), early/pregnant group (16% vs. 26%; *p* = 0.002) and late/non‐pregnant (45% vs. 37%; *p* = 0.05). Identifying eligible pregnant women was challenging due to restrictive enrolment criteria and competing studies, resulting in lower numbers in this group compared to the other groups. Contact with participants in between study visits for technical challenges with the adherence monitors occurred at least once in 89 (10%) participants, with a mean number of challenges of 2 (standard deviation 1.3) among those with any challenges.

Numerous demographic and socio‐behavioural differences were seen between the two settings (Table 1). The most notable differences included younger age, higher proportion of marriage, lower education, higher employment, higher perceived need for ART and more use of medications other than ART in Uganda. Additionally, structural barriers and food insecurity were higher in South Africa, as were probable depression, heavy alcohol use and cigarette use. Participant characteristics by group are shown in the Appendix (Table A1).

**Table 1:** Baseline participant characteristics (N = number; SD = standard deviation)

Characteristic	Uganda	South Africa	*p*‐Value
Enrollment (N)	483	421	–
Group (N, %)			0.004
Early/non‐pregnant	177 (36)	158 (38)	
Early/pregnant	129 (27)	76 (18)	
Late/non‐pregnant	177 (37)	187 (44)	
Median CD4 count (cells/μL, IQR)			
For participants > 350	469 (411, 593)	435 (387, 485)	<0.001
For participants < 200	123 (68, 164)	111 (51, 159)	0.07
Median viral load (log10 copies/mL, IQR)	4.3 (3.1, 4.9)	4.6 (4.0, 5.2)	<0.001
Female (N, %)	332 (69)	293 (70)	0.78
Mean age (years, SD)	31 (9)	34 (10)	<0.001
Married (N, %)	260 (55)	68 (16)	<0.001
Sexually active in the past six months (N, %)	392 (83)	375 (90)	0.005
Highest education (N, %):			<0.001
None/primary	188 (40)	56 (13)	
Secondary	284 (60)	364 (87)	
Literacy (N, %) in the local language (Runyankole or Xhosa)	405 (86)	371 (90)	0.086
Regular income (N, %)	60 (13)	192 (46)	<0.001
Employed (N, %)	392 (83)	179 (43)	<0.001
Exchange money for sex (N, %)	44 (9)	9 (2)	<0.001
Structural barrier score (mean score, SD)	3 (5)	13 (7)	<0.001
Instrumental support (mean score, SD)	30 (12)	31 (13)	0.82
Emotional support (mean score, SD)	34 (13)	32 (14)	0.03
Severe food insecurity (N, %)	140 (30)	284 (68)	<0.001
Stigma: perceived negative attitudes towards HIV (mean score, SD)	2 (2)	3 (2)	<0.001
Stigma: disclosure concerns (mean score, SD)	4 (3)	3 (3)	0.10
Disclosed aside from healthcare provider (N, %)	382 (81)	323 (77)	0.14
Disclosed outside of household (N, %)	254 (54)	147 (35)	<0.001
Necessity of ART (N, %)			<0.001
High	200 (42)	51 (12)	
Moderate	188 (40)	192 (46)	
Low	83 (18)	177 (42)	
Concern for ART (N, %)			<0.001
High	210 (45)	246 (59)	
Moderate	174 (37)	154 (37)	
Low	87 (18)	20 (5)	
Coping score (mean, SD)	2.2 (0.4)	2.4 (0.3)	<0.001
Medical mistrust (mean, SD)	1.9 (0.8)	2.1 (0.6)	<0.001
Conspiracy (mean, SD)	1.9 (0.7)	2.3 (0.6)	<0.001
Clinic satisfaction (mean, SD)	3.5 (0.4)	3.1 (0.4)	<0.001
First positive HIV test > 30 days prior to enrolment (N, %)	241 (51)	206 (54)	0.48
Use of medications other than ART (N, %)	386 (82)	85 (20)	<0.001
Physical well‐being (mean, SD)	39 (5)	42 (8)	<0.001
Mental well‐being (mean, SD)	46 (12)	38 (9)	<0.001
Probable depression (N, %)	114 (24)	207 (49)	<0.001
Heavy alcohol use (N, %)	41 (9)	119 (28)	<0.001
Cigarette use (N, %)	54 (12)	94 (22)	<0.001

ART, antiretroviral therapy; IQR, interquartile range. Data were missing for some characteristics as reflected in the percentage presented. Maximum missing data was <10%.

In Uganda, we found the following factors to be significantly associated with ART adherence: [positively] increasing age (*p* = 0.002), employment (*p* = 0.02), instrumental support (*p* < 0.001) and use of medications other than ART (*p* = 0.02); [negatively] sex work (*p* = 0.003), structural barriers (*p* = 0.006) and severe food insecurity (*p* = 0.03). In South Africa, we found the following factors to be significantly associated with ART adherence: [positively] increasing age (*p* = 0.04) and employment (*p* = 0.04); [negatively] instrumental support (*p* = 0.004), maladaptive coping (*p* = 0.03) and cigarette smoking (*p* = 0.01). We considered these factors as potential confounders of the relationship between HIV infection stage and ART adherence in the models testing our main study hypothesis (below).

Following enrolment, 29 participants were lost to follow up, 6 withdrew (due to participant preference or moving out of catchment) and 36 participants died (19 in Uganda, 17 in South Africa; *p* = 0.94). Most deaths (N = 30; 83%) occurred in participants initiating ART with late‐stage disease and five occurred before issuing the adherence monitor. Compared to participants who completed follow up, these participants had higher viral load. Fewer were married or employed. Structural barriers were higher and more had severe food insecurity. More had a recent diagnosis and fewer had used medications other than ART. They also had fewer disclosure concerns, lower clinic satisfaction, lower mental well‐being and more cigarette use (Appendix Table A2).

### 3.2 Adherence and viral suppression by setting

**Figure 2:**
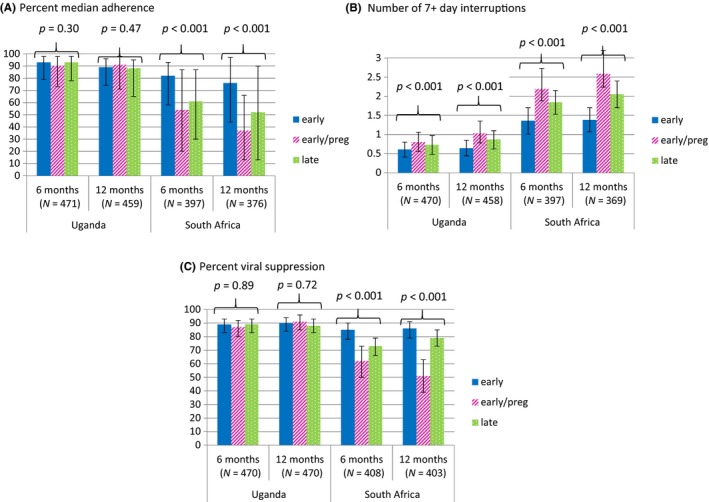
ART adherence and viral suppression among participants by study setting (Uganda vs. South Africa) and group: men and non‐pregnant women with early‐stage HIV infection (CD4 > 350 cells/μL), pregnant women with early‐stage HIV infection and men and non‐pregnant women with late‐stage HIV infection (CD4 < 200 cells/μL) **(A)** Percent median adherence, whereas **(B)** shows the number of 7+ day consecutive interruptions in adherence. **(C)** Percent viral suppression.

Technical function of the adherence monitors was documented for 97% of participant‐days of follow up (N = 288,804/298,398) (Figure 2). Viral suppression data were available for 895 participants (31 of whom did not contribute adherence data). In Uganda, median ART adherence at 12 months was 89% overall (interquartile range (IQR) 74% to 96%) and the median number of adherence interruptions of 7+ days was 0 (IQR 0 to 2). Viral suppression was 90% overall; 89%, 91% and 88% for the early/non‐pregnant, early pregnant and late/non‐pregnant groups respectively. No differences in overall adherence or viral suppression were seen among the three groups in this setting (all *p* > 0.72). However, more 7+ day interruptions were seen in the early/pregnant group versus early/non‐pregnant group (*p* = 0.004), and trended towards a difference with the late/non‐pregnant group (*p* = 0.06). In South Africa, median adherence at 12 months was 61% overall (IQR 26 to 86) and higher in the early/non‐pregnant group compared to the early/pregnant or late/non‐pregnant groups (76%, 37% and 52% respectively; *p* < 0.001 overall with *p* < 0.001 for 76% vs. 37%, *p* = 0.001 for 76% vs. 52% and *p* = 0.18 for 37% vs. 52%). Similar trends were seen in the number of adherence interruptions of 7+ days (1.4, 2.6 and 2.1 respectively; *p* = 0.001 overall with *p* = 0.001 for 1.4 vs. 2.6, *p* = 0.002 for 1.4 vs. 2.1 and *p* = 0.92 for 2.6 vs. 2.1) and viral suppression (86%, 51% and 79% respectively; *p* < 0.001 overall with *p* < 0.001 for 86% vs. 51%, *p* = 0.08 for 86% vs. 79% and *p* < 0.001 for 51% vs. 79%). Adherence was lower in months 0 to 6 versus months 7 to 12 in both settings with an estimated mean difference of −5.8 percentage points (pp) in Uganda (*p* < 0.001) and −13 pp in South Africa (*p* < 0.001). Results from the secondary data set accounting for device non‐use were nearly identical with no statistically significant differences.

We found similar adherence among the three groups in Uganda (*p* = 0.65) when controlling for the above‐noted potential confounders (Table 2). In South Africa, however, adherence was significantly higher among non‐pregnant individuals with early‐stage HIV infection compared to pregnant women with early‐stage infection and men and non‐pregnant women with late‐stage infection (−19.2 and −12.1 pp respectively; *p* < 0.001).

**Table 2:** Antiretroviral therapy (ART) adherence by HIV stage at ART initiation, stratified by country

Factor	Univariable findings		Multivariable findings^a^	
	Percentage point change (95% CI)	*p*‐Value	Percentage point change (95% CI)	*p*‐Value
Uganda				
Group				
Early/non‐pregnant	Ref	0.18	ref	0.65
Early/pregnant	−4.4 (−9.5, 0.7)	0.093	0.3 (−4.6, 5.2)	0.91
Late/non‐pregnant	−2.9 (−7.3, 1.5)	0.191	−1.7 (−5.5, 2.2)	0.40
South Africa				
Group				
Early/non‐pregnant	Ref	<0.001	ref	<0.001
Early/pregnant	−22.7 (−31.1, 14.4)	<0.001	−19.2 (−28.7, −9.7)	<0.001
Late/non‐pregnant	−13.3 (−19.8, −6.7)	<0.001	−12.1 (−18.7, −5.6)	<0.001

Values greater than zero indicate higher adherence; values less than zero indicate lower adherence.

^a^Controlling for age, employment, exchange of sex for money, structural barriers, instrumental support, severe food insecurity and use of medications other than ART in Uganda; and age, marital status, employment, structural barriers, instrumental support, perceived necessity of ART, physical well‐being and cigarette smoking in South Africa.

### 3.3 Predictors of adherence among non‐pregnant individuals starting ART with early‐stage HIV infection

In Uganda, adherence was significantly higher among non‐pregnant individuals starting ART with early‐stage HIV infection with increasing age (0.5 pp; *p* < 0.001) and lower with increasing structural barriers (−2.0 pp; *p* = 0.005) (Tables 3 and 4). In South Africa, adherence in this population was significantly higher with a regular income (6.7 pp; *p* = 0.04), higher perceived stigma (negative attitudes towards HIV; 1.9 pp; *p* = 0.04) and use of medications other than ART (9.8 pp, *p* = 0.005), but lower with maladaptive coping (−14.4 pp; *p* = 0.002) and cigarette smoking (−12.0 pp; *p* = 0.007).

**Table 3:** Socio‐behavioural factors influencing adherence among non‐pregnant individuals initiating antiretroviral therapy (ART) with early HIV infection in Uganda

Factor	Univariable findings		Multivariable findings	
	Percentage point change (95% CI)	*p*‐Value	Percentage point change (95% CI)	*p*‐Value
Female	−3.2 (−8.3, 2.0)	0.23	–	–
Age (increasing by year)	0.7 (0.4, 0.9)	<0.001	0.5 (0.3, 0.8)	<0.001
Married	2.2 (−2.4, 8.9)	0.25	–	–
Sexually active	−3.0 (−6.9, 0.91)	0.13	–	–
Education greater than primary	−2.9 (−8.6, 2.7)	0.31	–	–
Literacy in local language	−6.7 (−12.8, −0.7)	0.30	−4.0 (−9.5, 1.5)	0.16
Regular income	−1.8 (−6.4, 2.8)	0.45	–	–
Employed	11.1 (−1.7, 23.8)	0.09	0.3 (−7.7, 8.3)	0.95
Exchanging money for sex	2.7 (−3.6, 8.9)	0.40	–	–
Structural barriers score	−2.1 (−3.6, −0.7)	0.005	−2.0 (−3.3, −0.6)	0.005
Instrumental support	5.6 (−0.8, 12.0)	0.09	3.3 (−2.9, 9.4)	0.30
Severe food insecurity	−3.2 (−7.3, 1.0)	0.13	–	–
Stigma: perceived negative attitudes towards HIV	−0.6 (−1.7, 0.5)	0.30	–	–
Stigma: disclosure concerns	−0.1 (−0.8, 0.7)	0.80	–	–
Disclosed aside from healthcare provider	4.8 (−1.9, 11.6)	0.16	–	–
Disclosed outside of household	3.6 (−1.3, 8.5)	0.15	–	–
Perceived necessity of ART	1.3 (−3.1, 5.7)	0.57	–	–
Perceived concern for ART	−0.7 (−4.3, 2.9)	0.70	–	–
Maladaptive coping	−0.7 (−5.3, 3.9)	0.76	–	–
Medical mistrust	0.2 (−2.0, 2.4)	0.86	–	–
Conspiracy	0.7 (−1.5, 2.9)	0.52	–	–
Clinic satisfaction	1.7 (−2.8, 6.1)	0.46	–	–
First positive HIV test > 30 days before enrolment	2.7 (−3.1, 8.4)	0.36	–	–
Use of medications other than ART	14.0 (−8.8, 36.9)	0.23	–	–
Physical well‐being	0.1 (−0.2, 0.2)	0.93	–	–
Mental well‐being	0.1 (−0.1, 0.3)	0.26	–	–
Probable depression	0.4 (−3.8, 4.7)	0.84	–	–
Heavy alcohol use	−2.7 (−8.4, 3.0)	0.35	–	–
Smoke cigarettes	−1.0 (−7.3, 5.4)	0.76	–	–

Values greater than zero indicate higher adherence; values less than zero indicate lower adherence.

**Table 4:** Socio‐behavioural factors influencing adherence among non‐pregnant individuals initiating antiretroviral therapy (ART) with early HIV infection in South Africa

Factor	Univariable findings		Multivariable findings	
	Percentage point change (95% CI)	*p*‐Value	Percentage point change (95% CI)	*p*‐Value
Female	−4.3 (−14.1, 5.6)	0.40	–	–
Age (increasing by year)	0.2 (−0.2, 0.7)	0.28	–	–
Married	6.6 (−4.7, 17.9)	0.25	–	–
Sexually active	−2.1 (−11.9, 7.6)	0.67	–	–
Education greater than primary	−0.9 (−12.0, 10.1)	0.87	–	–
Literacy in local language	−2.0 (−18.5, 14.6)	0.82	–	–
Regular income	6.3 (0.44, 12.2)	0.035	6.7 (1.0, 12.4)	0.04
Employed	3.7 (−5.6, 13.0)	0.44	–	–
Exchanging money for sex	−6.0 (−62.8, 50.8)	0.84		
Structural barriers scale	−0.5 (−1.0, −0.1)	0.015	−0.5 (−0.9, −0.1)	0.06
Instrumental support	1.1 (−6.8, 9.0)	0.78	–	–
Severe food insecurity	3.9 (−1.7, 9.5)	0.17	–	–
Stigma: perceived negative attitudes towards HIV	1.5 (−0.2, 3.3)	0.090	1.9 (0.1, 3.7)	0.04
Stigma: disclosure concerns	−0.3 (−1.7, 1.2)	0.73	–	–
Disclosed aside from healthcare provider	2.2 (−10.2, 14.6)	0.73	–	–
Disclosed outside of household	2.7 (−6.6, 12.0)	0.57	–	–
Perceived necessity of ART	4.7 (−3.9, 13.3)	0.30	–	–
Perceived concern for ART	−1.6 (−9.6, 6.3)	0.69	–	–
Maladaptive coping	−15.0 (−26.0, −4.0)	0.007	−14.4 (−23.7, −5.2)	0.002
Medical mistrust	2.9 (−4.6, 10.3)	0.45	–	–
Conspiracy	−0.1 (−7.3, 7.2)	0.99	–	–
Clinic satisfaction	4.7 (−1.1, 10.5)	0.11	–	–
First positive HIV test > 30 days before enrolment	−3.7 (−14.3, 6.9)	0.50	–	–
Use of medications other than ART	12.2 (4.8, 19.6)	0.001	9.8 (2.9, 16.7)	0.005
Physical well‐being	−0.5 (−0.8, −0.1)	0.011	−0.2 (−0.5, 0.2)	0.38
Mental well‐being	0.2 (−0.1, 0.5)	0.13	–	–
Probable depression	−5.5 (−12.0, 1.0)	0.10	–	–
Heavy alcohol use	−11.3 (−19.7, −2.9)	0.009	−5.6 (−13.3, 2.1)	0.15
Smoke cigarettes	−12.2 (−22.2, −2.2)	0.017	−12.0 (−20.7, −3.2)	0.007

Values greater than zero indicate higher adherence; values less than zero indicate lower adherence.

## 4 Discussion

In this large, observational cohort study of individuals enrolled in routine HIV care in Uganda and South Africa, we found that electronically monitored adherence and viral suppression among individuals initiating ART with early‐stage HIV infection were as high or higher than those initiating ART with late‐stage HIV infection. In Uganda, adherence was generally high and did not differ by stage of ART initiation or pregnancy, although adherence interruptions of 7+ days were higher in pregnant women; viral suppression achieved the UNAIDS goal of 90% at 12 months. In South Africa, overall adherence was much lower and adherence interruptions of 7+ days were more common in pregnant women and those with late‐stage HIV infection compared to early‐stage HIV infection. Viral suppression in South Africa reached the UNAIDS goal in individuals initiating ART with early‐stage HIV infection, but not in the other groups.

Our study adds important effectiveness data from publicly operated HIV programs in sub‐Saharan Africa. The high levels of viral suppression among individuals with early‐stage HIV infection in our study are consistent with both the multi‐national START trial and the TEMPRANO trial in Côte d/Ivoire, which demonstrated the personal health benefits of early ART initiation 5, 37. In START, viral suppression at 12 months was 98% among individuals with CD4 counts >500 cells/μL who started ART immediately and 97% among those who waited to initiate ART (i.e. until the CD4 count dropped <350 cells/μL or they developed AIDS or another condition requiring ART). In TEMPRANO, viral suppression at 12 months was lower overall, but still >80% for all participants regardless of timing of ART initiation. Data, however, were missing for 13% of participants reaching 12 months of follow up. Adherence was not reported in either study, but was likely high given the known relationship between adherence and viral suppression. Unlike these trials, our findings reflect observation of routine clinical care with only three study visits occurring over one year for approximately 90% of participants. While adherence monitoring could have acted as an adherence intervention (i.e. the Hawthorne effect), our results bode well for individuals initiating ART with high CD4 counts outside of research studies. A recent trial of streamlined clinical care in Uganda and Kenya, the SEARCH trial, also found high levels of viral suppression among individuals with high CD4 counts, although adherence again was not assessed directly 38.

While overall adherence was high among non‐pregnant individuals starting ART with early‐stage HIV infection, some struggled. The predictor analysis showed lower adherence was associated with younger age and structural barriers in Uganda, and no regular income, lower stigma, maladaptive coping, lack of other medication use and cigarette smoking in South Africa. As global HIV policy increasingly focuses on asymptomatic PLWH, many of these factors can serve as markers of individuals at risk of low adherence who should be carefully screened for enhanced counselling, education and other available interventions. Novel interventions directed at structural barriers, stigma and maladaptive coping should also be explored.

Interestingly, social support was not a significant factor, refuting our hypothesis. The impact of social support may have been limited because we studied individuals engaged in care; those able to enter care may have access to a basic level of social support. Additionally, disclosure rates were relatively high and stigma relatively low, suggesting possible normalization of HIV care among the populations studied (consistent with global trends 39). That said, the association between adherence and perceived stigma in South Africa suggests stigma avoidance as a motivating factor; persistent disclosure concerns have also been observed in another recent South Africa study 40. An additional unexpected finding was the lack of effect for beliefs about ART (i.e. necessity and concern). These factors may have less impact if social norms promote taking ART and should be explored in future analyses.

In contrast to the encouraging findings among non‐pregnant women and men with early HIV infection, the low adherence and viral suppression among pregnant women with high CD4 counts in South Africa are concerning. As presented elsewhere 41, baseline factors associated with greater adherence among female participants in South Africa included incident (vs. prevalent) pregnancy, better coping, belief in the need for ART and lack of cigarette smoking. A full manuscript detailing the relationships between pregnancy and adherence and viral suppression at the two study sites, as well as implications for potential interventions, is in progress. Rollout of universal lifelong therapy for pregnant and breastfeeding women (i.e. Option B+) has been shown to increase ART uptake and initial indications of retention are reasonably high with 77% in care in Malawi 42. However, loss‐to‐follow‐up rates have been as high as 58% when women start ART at large clinics on the day of HIV diagnosis 43. Similar findings have been reported in Mozambique where loss‐to‐follow‐up rates are 38% among women initiating ART with CD4 counts >350 cells/μL 44. In contrast, data from another South African study found viral suppression rates of 74% at 12 months for women with same day initiation of ART with Option B+ 45. Together, these findings call for further research and interventions to identify and support women struggling during this vulnerable time.

Similarly, moderate levels of adherence and viral suppression among individuals with late‐stage HIV infection warrants continued support for this population. A meta‐analysis showed CD4 counts at presentation to care and ART initiation in sub‐Saharan Africa did not increase between 2003 and 2013 19. Additional research is needed to see the impact of WHO recommendations on universal ART access; however, attention is clearly needed to identify individuals living with HIV earlier and support them to access treatment.

Our study has many strengths including detailed adherence data, thorough socio‐behavioural metrics, large sample size, diverse clinical settings, observation of routine clinical care provision and high retention (approximately 90% for all groups). Limitations include potential misclassification of adherence, principally due to device non‐use. The relatively higher degree of viral suppression with lower levels of adherence in South Africa could suggest more device non‐use in that setting. Technical malfunction with the devices and/or cellular transmission of data may have also contributed to misclassification of adherence, although adherence data were available for 97% of participant‐days and no technical challenges were observed for 90% of participants. Additionally, we only followed individuals for 12 months. An estimated one‐third of individuals fail therapy at two years 46 and the longitudinal impact of stage of disease at treatment initiation remains unknown. Finally, 17% of screened individuals declined to participate in the study. If these individuals were less likely to achieve high adherence, differential selection could have biased our estimates of adherence upward.

## 5 Conclusions

In sum, our study shows promise that adherence is high in most non‐pregnant individuals initiating ART with early HIV disease, thus supporting the universal roll out of ART. However, adherence interventions may be needed for youth and those with no regular income, no prior experience taking medication, structural barriers, maladaptive coping skills and cigarette use; adherence trends should also be assessed with longer follow up. The challenges seen among pregnant women and those entering care late in South Africa call for differentiated care models (i.e. provision of services that are tailored to the needs of a given individual or sub‐population to improve outcomes and efficiencies within health systems). While modelling data suggest an AIDS‐free generation may be possible and community‐based “fast‐track” ART initiation can achieve high rates of virological suppression 47, recent data on reducing HIV incidence has been disappointing 48, 49. More data are needed to understand the mechanisms behind these findings and the role adherence plays, particularly among vulnerable populations.

## Competing interests

JEH has received consultation fees from Merck. All other authors have no competing interests to declare.

## Authors’ contributions

JEH, CO, MBB, DRB, MJS, LTM and ACT designed the study. JEH, CO, MBB, SA and GA led the study. NM and JEH conducted the analyses with input from CO, DRB, MJS, LTM, ACT, ITK and BHG. KB, AK, SM, VK, NK and AC contributed to the design of the study and oversaw the data collection. JEH wrote the first draft of the manuscript. All authors contributed to the execution of the work presented in the manuscript, and all critically reviewed and approved the final version.

## Funding

Bill and Melinda Gates Foundation (OPP1056051). The study funder had no role in study design, data gathering, analysis and interpretation, or writing of the report. The corresponding author had full access to all study data and had final responsibility for the decision to submit for publication.

## Appendix 1

### 1.1

**Table A1:** Participant characteristics by country and group

	Early/non‐pregnant			Early/pregnant			Late/non‐pregnant		
Characteristic	Uganda (N = 177)	South Africa (N = 158)	*p*‐Value	Uganda (N = 129)	South Africa (N = 76)	*p*‐Value	Uganda (N = 177)	South Africa (N = 187)	*p*‐Value
Median CD4 count (cells/μL, IQR)	437 (395, 486)	423.5 (385, 465)	0.02	574 (454, 699)	486 (408, 675)	0.03	122.5 (68, 164)	111 (51, 159)	0.06
Median viral load (log10 copies/mL, IQR)	3.9 (2.8, 4.5)	4.4 (3.7, 4.8)	<0.001	3.5 (2.2, 4.2)	4.1 (3.4, 4.5)	<0.001	5.0 (4.5, 5.4)	5.1 (4.6, 5.5)	0.06
Female (N, %)	118 (67)	114 (72)	0.28	129 (100)	76 (100)	–	85 (48)	103 (55)	0.18
Mean age (years, SD)	33 (10)	35 (11)	0.02	26 (6)	26 (4.6)	0.98	33 (8)	36 (9.6)	0.004
Married (N, %)	86 (49)	30 (19)	<0.001	101 (81)	5 (7)	<0.001	73 (42)	33 (18)	<0.001
Sexually active in the past six months (N, %)	142 (81)	139 (89)	0.06	125 (100)	74 (99)	0.20	125 (73)	162 (87)	0.001
Highest education (N, %)			<0.001			<0.001			<0.001
None/primary	81 (46)	24 (15)		34 (27)	4 (5)		73 (42)	28 (15)	
Secondary	94 (54)	134 (85)		91 (73)	71 (95)		99 (58)	159 (85)	
Literacy (N, %) in the local language(Runyankole or Xhosa)	152 (87)	139 (90)	0.43	114 (91)	71 (96)	0.21	139 (81)	161 (87)	0.11
Regular income (N, %)	24 (14)	83 (53)	<0.001	15 (12)	25 (33)	<0.001	21 (12)	84 (45)	0.001
Employed (N, %)	158 (90)	79 (50)	<0.001	84 (67)	22 (29)	<0.001	150 (87)	78 (42)	<0.001
Exchange money for sex (N, %)	16 (9)	2 (1)	<0.006	20 (16)	2 (3)	0.004	8 (5)	5 (3)	0.31
Structural barrier score (mean score, SD)	4 (6)	13 (7)	<0.001	2 (5)	15 (7)	<0.001	1 (4)	13 (6)	<0.001
Instrumental support (mean score, SD)	32 (11)	31 (12)	0.29	28 (13)	31 (13)	0.19	30 (11)	32 (14)	0.29
Emotional support (mean score, SD)	35 (12)	32 (13)	0.03	32 (14)	32 (13)	0.88	35 (13)	33 (14)	0.19
Severe food insecurity (N, %)	64 (37)	101 (64)	<0.001	30 (24)	50 (67)	<0.001	46 (27)	133 (71)	<0.001
Stigma: perceived negative attitudes (mean score, SD)	1.6 (1.8)	2.7 (1.9)	<0.001	2.2 (2.0)	1.9 (1.7)	0.23	1.8 (1.9)	2.7 (1.8)	<0.001
Stigma: disclosure concerns (mean score, SD)	3.5 (2.5)	3.2 (2.5)	0.39	4.4 (2.6)	3.7 (2.6)	0.08	3.3 (2.4)	3.3 (2.5)	0.84
Disclosed aside from healthcare provider (N, %)	141 (81)	133 (84)	0.39	93 (74)	39 (53)	0.002	148 (87)	151 (81)	0.14
Disclosed outside of household (N, %)	112 (64)	74 (47)	0.002	50 (40)	15 (20)	0.004	92 (54)	58 (31)	<0.001
Necessity of ART (N, %)			<0.001			<0.001			<0.001
Low	31 (18)	64 (41)		21 (17)	40 (53)		31 (18)	73 (39)	
Moderate	68 (39)	77 (49)		48 (38)	26 (35)		72 (42)	89 (48)	
High	76 (43)	17 (11)		56 (45)	9 (12)		68 (40)	25 (13)	
Concern for ART (N, %)			0.004			<0.001			<0.001
Low	20 (11)	6 (4)		33 (26)	3 (4)		34 (20)	11 (6)	
Moderate	72 (41)	53 (34)		29 (23)	28 (37)		73 (43)	73 (39)	
High	83 (47)	99 (63)		63 (50)	44 (59)		64 (37)	103 (55)	
Coping score (mean, SD)	2.2 (0.4)	2.4 (0.3)	<0.001	2.2 (0.4)	2.3 (0.2)	0.18	2.2 (0.4)	2.4 (0.3)	0.001
Medical mistrust (mean, SD)	1.9 (0.8)	2.2 (0.5)	<0.001	1.8 (0.8)	2.0 (0.6)	0.04	2.0 (0.8)	2.1 (0.6)	0.04
Conspiracy (mean, SD)	1.9 (0.7)	2.3 (0.5)	<0.001	1.8 (0.7)	2.2 (0.4)	<0.001	1.9 (0.6)	2.3 (0.6)	<0.001
Clinic satisfaction (mean, SD)	3.5 (0.4)	3.1 (0.5)	<0.001	3.5 (0.5)	3.2 (0.5)	<0.001	3.5 (0.4)	3.1 (0.4)	<0.001
First positive HIV test >30 days prior to enrollment (N, %)	114 (65)	97 (69)	0.47	64 (51)	21 (29)	0.003	63 (37)	88 (51)	0.007
Use of medications other than ART (N, %)	157 (90)	29 (18)	<0.001	100 (80)	3 (4)	<0.001	129 (75)	53 (28)	<0.001
Physical well‐being (mean, SD)	40 (5)	44 (8)	<0.001	41 (5)	45 (7)	<0.001	37 (5)	40 (8)	0.0015
Mental well‐being (mean, SD)	47 (11)	39 (9)	<0.001	49 (10)	37 (8)	<0.001	42 (13)	38 (10)	0.0037
Probable depression (N, %)	35 (20)	81 (51)	<0.001	26 (21)	37 (49)	<0.001	53 (31)	89 (48)	0.001
Heavy alcohol use (N, %)	16 (9)	50 (32)	<0.001	8 (6)	14 (19)	0.007	17 (10)	55 (29)	<0.001
Cigarette use (N, %)	20 (11)	38 (24)	0.002	2 (2)	9 (12)	0.002	32 (19)	47 (25)	0.15

ART, antiretroviral therapy; IQR, interquartile range. Data were missing for some characteristics as reflected in the percentage presented. Maximum missing data was <10%

**Table A2:** Baseline participant characteristics among individuals who completed versus did not complete the study due to loss to follow up, disenrollment or death

Characteristic	Completed	Lost/disenrolled/died	*p*‐Value
Enrollment (N)	805	99	–
Group			0.06
Early/non‐pregnant	308 (38)	27 (27)	
Early/pregnant	183 (23)	22 (22)	
Late/non‐pregnant	314 (39)	50 (51)	
Median CD4 count (cells/μL)			
For participants >350	451 (399–526)	458 (412–528)	0.35
For participants <200	120 (60–160)	90 (50–150)	0.18
Median viral load (log_10_ copies/mL, IQR)	4.4 (3.5, 5.0)	4.7 (4.2, 5.2)	0.004
Female (N, %)	557 (69)	68 (69)	0.92
Mean age (years, SD)	33 (10)	32 (8)	0.54
Married (N, %)	311 (39)	17 (20)	<0.001
Sexually active (N, %)	698 (87)	69 (80)	0.09
Highest education (N, %)			0.48
None/primary	223 (28)	21 (24)	
Secondary	582 (72)	66 (76)	
Literacy (N, %) in the local language (Runyankole or Xhosa)	706 (88)	70 (81)	0.07
Regular income (N, %)	224 (28)	28 (32)	0.39
Employed (N, %)	529 (66)	42 (48)	0.001
Exchanging money for sex (N, %)	49 (6)	4 (5)	0.14
Structural barrier score (mean score, SD)	8 (8)	9 (8)	0.03
Instrumental support (mean score, SD)	30 (12)	33 (13)	0.09
Emotional support (mean score, SD)	33 (14)	35 (13)	0.39
Severe food insecurity (N, %)	373 (46)	51 (59)	0.02
Stigma: perceived negative attitudes (mean score, SD)	2.2 (1.9)	2.3 (2.0)	0.67
Stigma: disclosure concerns (mean score, SD)	3.6 (2.5)	3.0 (2.5)	0.03
Disclosed aside from healthcare provider (N, %)	635 (79)	70 (81)	0.60
Disclosed outside of household (N, %)	362 (45)	39 (45)	0.95
Perceived necessity of ART (N, %)			0.47
High	231 (29)	29 (34)	
Moderate	343 (43)	37 (43)	
Low	231 (29)	20 (23)	
Perceived concern for ART (N, %)			0.09
High	101 (13)	6 (6)	
Moderate	288 (36)	40 (47)	
Low	416 (52)	40 (47)	
Maladaptive coping (mean, SD)	2.3 (0.3)	2.3 (0.4)	0.37
Medical mistrust (mean, SD)	2.0 (0.7)	2.1 (0.6)	0.62
Conspiracy (mean, SD)	2.1 (0.7)	2.2 (0.6)	0.09
Clinic satisfaction (mean, SD)	3.3 (0.5)	3.2 (0.4)	0.02
First positive HIV test > 30 days prior to enrolment (N, %)	415 (54)	32 (39)	0.01
Use of medications other than ART (N, %)	435 (54)	36 (41)	0.025
Physical well‐being (mean, SD)	41 (7)	41 (8)	0.95
Mental well‐being (mean, SD)	42 (11)	38 (11)	0.003
Probable depression (N, %)	283 (35)	38 (44)	0.10
Heavy alcohol use (N, %)	141 (18)	19 (22)	0.30
Cigarette use (N, %)	127 (16)	21 (24)	0.04

ART, antiretroviral therapy; IQR, interquartile range. Data were missing for some characteristics as reflected in the percentage presented. Maximum missing data was <10%
